# Psoriatic Arthritis: Developments in 2025

**DOI:** 10.31138/mjr.200925.per

**Published:** 2026-01-08

**Authors:** Christina Spanopoulou, Pelagia Katsimbri

**Affiliations:** Department of Rheumatology, KAT Hospital, Athens, Greece

**Keywords:** psoriatic arthritis, incidence, aetiology, antirheumatic agents, treatment outcome, disease management

## Abstract

Psoriatic arthritis (PsA) a complex, multifaceted autoimmune disease involving the skin and joints, presents a diagnostic and therapeutic challenge due to its heterogenous nature and existence of comorbidities. Early diagnosis is essential to prevent functional disability and achieve disease remission. The previous year has seen further advancements in the better understanding of PsA that include identifying patients at risk and the phases of transition from psoriasis (PsO) to PsA as well as new insights in pathogenesis and the development of newer treatments and therapeutic protocols. These developments are paving the way for more individualised management protocols aiming for further improvement in the quality of life for patients living with PsA. In this narrative review article, we reviewed the literature from major scientific databases for PsA from June 2024 to September 2025.

## INTRODUCTION

Psoriatic arthritis (PsA) is a complex, multifaceted autoimmune disease involving the skin and joints. The development of PsA involves a complex interplay of genetic predisposition, environmental factors and immunological dysregulation. The multifaceted nature of the disease is seen through the coexistence of extra-musculoskeletal inflammation and comorbidities such as inflammatory bowel disease (IBD), uveitis, metabolic syndrome, depression, and cardiovascular disease.^1–3^

The previous year has seen advancements in the understanding of PsA that have looked at disease progression and the phases of transition from PsO to PsA including identifying individuals at risk. Apart from new treatments, there were also advances in genetics and immunopathogenesis with new insights in the role of T cells and cytokines that have led to the development of targeted therapies as well as refining and developing tools and biomarkers to identify PsA earlier as late diagnosis leads to worse outcomes. In this narrative review, an electronic literature search was performed for PsA from June 2024 to September 2025. Databases, including Medline/PubMed and Scopus, were used to identify relevant literature using key terms: “Psoriatic Arthritis”, “Incidence”, “Etiology”, “Antirheumatic Agents”, “Treatment Outcome”, “Disease Management”. We provide an up-to-date overview of the past year, with a focus on emerging insights into treatment and management.

## INCIDENCE

PsA is equally present in men and women affecting around 30% of patients with PsO. Understanding the incidence and timing of PsA diagnosis can help in developing screening strategies and prediction tools in primary care PsO populations. As in previous assessments PsA annual incidence was confirmed by the UK TUDOR Trial at 2.2/100patient years over a 2-year period.^4^ A trial using data from the National Health and Nutrition Examination Survey (NHANES) database and novel risk assessment models integrating parameters beyond the traditional skin lesions and joint symptoms showed key predictors for PsA development in PsO populations were age, education, fasting glucose, and comorbidities such as hypertension, thyroid disease, and chronic bronchitis.^5^

Even though PsA affects men and women equally, emerging evidence suggests that gender influences disease characteristics and response to treatment. A recent systematic literature review demonstrated women exhibiting higher disease activity, greater pain perception and lower response rates to biologics.^6^ Complementing this, a Danish study found men achieving better clinical responses to Tumour Necrosis Factor inhibitors (TNFi), with longer treatment persistence compared to women.^7^ These findings highlight the potential for individualised therapeutic strategies, supporting the need for treatment selection to be guided by gender alongside clinical and immunological factors.

## PATHOGENESIS

Although PsA and PsO share several common genetic variants influencing immune pathways, PsA also exhibits distinct gene expression patterns. These genetic differences help explain the divergent responses to biologic treatments observed between skin and joint disease.^8^

Observational and genetic studies have indicated associations between PsA and other autoimmune diseases, though causality is largely undetermined. Mendelian randomisation studies recently identified conditions such as Crohn’s disease, Hashimoto thyroiditis, systemic lupus erythematosus, rheumatoid arthritis, ankylosing spondylitis and vitiligo to increase PsA risk. Mendelian randomisation studies also found PsA risk to be associated with specific plasma proteins such as IL-10 and apolipoprotein F. Regular monitoring is essential for patients with psoriatic disease and other autoimmune diseases, as several plasma proteins have also been linked to PsA development.^9,10^

The IL-23/IL-17 axis is pivotal in the pathogenesis of PsA.^11^ Activated T cells from the skin, joints and intestine differentiate into tissue-resident memory T cells (TRM) that are now recognised as heterogeneous and plastic. After antigen recognition or IL-23 stimulation, TRMs become reactivated, adopt a pro-inflammatory effector phenotype and can even leave the tissue entering the circulation, potentially seeding inflammation at distant sites.^12–14^ As a result, populations of CD4+, CD8+, TH1 and TH17 lymphocytes expand under the influence of IL-23 and IL-12 producing TNF, IL-17 and other cytokines, chemokines and metalloproteases. The circulating TRMs suggest they are capable of retrograde migration and could serve as biomarkers of disease activity or result in resistance to therapy. Importantly, for durable remission in PsO complete suppression of TRM is necessary. IL-17A inhibition reduces TRM-specific markers more rapidly than TNF inhibition or methotrexate, but IL-23 blockade appears to more effectively deplete pathogenic TRM markers enriched in the psoriatic epidermis. Such differences may explain why IL-23 inhibition is associated with longer remission and possibly fewer relapses than IL-17 blockade in PsO and early PsA.^15,16^

The gut microbiome is a key player in PsA pathogenesis as illustrated by more PsA patients than healthy controls having an affected microbiome, and the growing body of evidence suggesting that intestinal dysbiosis and impaired barrier function contribute to systemic immune dysregulation. Dysbiosis in PsA is characterised by reduced microbial diversity and depletion of anti-inflammatory commensal bacteria and potentially pro-inflammatory taxa.^17^ Similar microbial signatures are observed in Crohn’s disease, where they have been consistently linked to impaired anti-inflammatory responses and heightened intestinal inflammation.^18^ The recent detection of microbial DNA and bacterial products in the bloodstream and synovial fluid of PsA patients support the concept of bacterial translocation, providing a link between gut and joint inflammation.^19^ Beyond changes in microbial composition, dysbiosis affects host immunity through bacterial metabolites. Short-chain fatty acids (SCFAs), produced by the fermentation of dietary fibre by gut bacteria, help maintain the intestinal barrier, stimulate IgA responses and promote regulatory T-cell differentiation. Loss of SCFA-producing bacteria in PsA is associated with impaired mucosal defence, oxidative stress and Th17-driven inflammation.^20^ Similarly, bile acids (BAs), modified by gut microbes, regulate the balance between Th17 and Treg cells and influence systemic immune responses. Reduced serum levels of secondary BAs have been linked to a higher risk of PsO to PsAprogression, while BA supplementation may benefit selected patients.^21^ Also, gut bacteria metabolise dietary tryptophan into derivatives, which act on the aryl hydrocarbon receptor (AHR) to modulate mucosal and systemic immunity. Early trials of AHR agonists have demonstrated clinical efficacy in psoriatic disease.^22^ The gut microbiota-derived metabolite, trimethylamine N-oxide (TMAO), is elevated in PsO patients correlating with cardiovascular risk and disease activity, further highlighting the link between microbial metabolism, systemic inflammation, and comorbidities.^23^ Experimental models also support the gut–joint axis theory. Germ-free mice remain resistant to arthritis development, whereas colonisation with certain bacteria promotes synovial inflammation.^24^ Current translational studies in humans have demonstrated differences between PsA, PsO and healthy controls through integrated metagenomic and metabolomic profiling, revealing distinct patterns in gut microbial composition, reduced short-chain fatty acid levels and elevated inflammatory biomarkers such as faecal calprotectin.^25^ These insights are promising therapeutically. Western diet and obesity contribute to dysbiosis and PsA risk. Present day studies on probiotics, prebiotics and SCFA supplementation offer possible strategies to restore microbial balance and immune tolerance.^26^ More experimental interventions such as faecal micro-biota transplantation (FMT) and bacteriophage therapy in PsA have not demonstrated efficacy in PsA.^27^ Notably, disease-modifying antirheumatic drugs (DMARDs) may indirectly alter microbiota composition and gut permeability, while paradoxical responses to antibiotics and anti-TNF therapy underscore the complexity of microbiome–immune interactions.^28^ Collectively, these findings support PsA as a systemic disorder rooted in the gut–immune axis rather than a disease confined to the joints. Future research is needed to validate possible biomarkers and microbiome-targeted interventions for PsA.^29^

### PsO to PsA transition

Early disease intervention is important if we are to succeed in achieving disease remission. Identifying and defining the various stages of PsA is important. EULAR have defined 3 stages in PsO to PsA transition based on clinical signs and symptoms. The first stage includes high risk patients for PsA, such as those with severe skin PsO, nail involvement or obesity. The next stage is PsO patients with subclinical PsA when they start experiencing arthralgias or signs of synovio-entheseal inflammation on imaging without clinical synovitis. Finally, clinical PsA is patients fulfilling CASPAR criteria.^30^ However, differentiating early or very early PsA from subclinical PsA is important. Firstly, so the right treatment is given depending on the extent of disease involvement and secondly to define a window of opportunity to modify the natural history of the disease. Recent proposals have expanded PsA development into three stages beyond the subclinical phase, where subclinical PsA involves synovio-entheseal symptoms without imaging or clinical evidence of inflammation. Very early PsA adds imaging evidence of inflammation without clinical arthritis or enthesitis. Clinical arthritis or enthesitis of less than 2 years defines early PsA, while established PsA refers to disease of more than 2 years, fulfilling CASPAR criteria. These definitions are better for developing individualised algorithms for the management of PsA that consider risk factors, clinical and molecular characteristics and imaging patterns.^31^

## TREATMENT

The heterogenous nature of PsA makes treatment selection challenging. Novel agents and preventative trial designs are rapidly evolving the therapeutic landscape. Central to these advances are innovations in cytokine targeting, oral therapies, real-world evidence, combination strategies and disease interception.

### Tumour Necrosis Factor inhibitors

The first biologic DMARDS (bDMARDs) used in PsA management were TNFis. All are effective across all PsA domains and have also demonstrated inhibition or reduction of radiographic progression, with safety established in both the short and long term. Even though a recent meta-analysis showed a similar efficacy for ACR 20 response between adalimumab, etanercept, and infliximab, etanercept is less immunogenic because it is not a monoclonal antibody and doesn’t require concomitant administration with methotrexate to maintain its long-term effectiveness.^32^ However, etanercept is less effective than the monoclonal TNFi in the treatment of extra-musculoskeletal PsA manifestations, such as uveitis or IBD.^33^ A current Cochrane systematic review assessing benefits and harms of TNFi in PsA showed moderate evidence for substantial improvement, lower disease activity, small decrease in radiographic progression and better quality of life in TNF-treated conventional DMARD-inadequate responders (csDMARD-IR). Despite the report’s limitations, there was evidence for improvement in physical function with no differences to placebo in serious adverse events.^34^

### IL-17 inhibitors (IL-17i)

Inhibiting the IL-17/IL-23 pathway remains a cornerstone of PsA therapy. Secukinumab, the first approved IL-17A inhibitor for PsA showed efficacy for peripheral arthritis, spondylitis, dactylitis, enthesitis, and skin and nail disease. Nevertheless, in the first head-to-head clinical trial in PsA, secukinumab showed superiority to TNFi (adalimumab) for the skin but not for arthritis. Following improvements seen for the key clinical disease domains in the FUTURE 1 trial with an intravenous (IV) loading dose of secukinumab, the recently published INVIGORATE-2 trial looked at the efficacy, safety and tolerability of IV secukinumab in PsA to provide additional flexibility and individualised treatment options. All primary and secondary endpoints were met with IV secukinumab with sustained or increased improvements in disease measures up to week 52.^35^

Ixekizumab also selectively targets IL-17A with efficacy in all clinical PsA domains as well as inhibition of radiographic progression. Ixekizumab has also demonstrated superiority versus TNFi (adalimumab) for the composite primary endpoint of ACR50 plus PASI 100 response at week 24, but with comparable results with adalimumab for the musculoskeletal domain.^36^ A current post hoc analysis of the SPIRIT H2H trial looking at simultaneous distal interphalangeal (DIP) joint involvement and nail PsO confirmed the synovial-entheseal complex theory that DIP joint involvement is almost always adjacent to nail PsO, with ixekizumab showing greater resolution of DIP joint tenderness, swelling and resolution of nail PsO than adalimumab. These findings support previous superiority of ixekizumab versus comparators for nail PsO and extend this superiority to the DIP-nail musculoskeletal domain.^37^

Bimekizumab, the latest IL-17i approved for PsA inhibits both IL-17A and IL-17F. The BE ACTIVE, BE OPTIMAL and BE COMPLETE studies demonstrated efficacy in both joints (ACR50) and skin (PASI100), whilst a recent post hoc analysis of the BE OPTIMAL and BE COMPLETE studies looking at patient-reported outcomes (PROs) and health-related quality of life (HRQoL), showed rapid improvements in all PROs after only 4 weeks and just one dose, adding further evidence for the efficacy of bimekizumab for both clinical symptoms and PROs.^38^ A further study looking at the effect of bimekizumab on patient-reported disease impact using the 12-item PsA Impact of Disease (PsAID-12) questionnaire confirmed its rapid and sustained clinically meaningful improvements.^39^ Further evidence showed these improvements were sustained up to 1 year in half of all patients with substantial improvement in the HRQoL scores and overall work production.^40^ Long term safety data with a total bimekizumab exposure of >2500 patient-years for PsA cohorts, showed a consistent and favourable safety profile with overall incidence rates of treatment-emergent adverse events (TEAEs) being comparable in all cohorts and remaining stable over extended periods of treatment with no new safety signals.^41^

Newer IL-17i for psoriatic diseases such as izokibep and sonelokimab have optimised molecular design. Izokibep, an affibody-based IL-17A inhibitor, demonstrated robust efficacy in phase 2 trials, achieving rapid and sustained ACR50 and PASI responses with favourable tolerability. Its small size and albumin-binding domain enhance tissue penetration and extend the plasma half-life, offering advantages over conventional monoclonal antibodies. However, domain-specific analyses, such as enthesitis resolution, revealed no superiority over existing IL-17i, illustrating the challenge of translating molecular potency into broad clinical benefit.^42^ Similarly, sonelokimab, derived from alpaca nanobody technology, offers a trivalent construct targeting IL-17A/A, IL-17A/F and IL-17F/F, with an albumin-binding domain that improves pharmacokinetics. In the phase 2 ARGO trial, sonelokimab achieved significant ACR50 improvements progressing its development into the IZAR phase 3 trial in PsA to further evaluate efficacy and safety outcomes.^43,44^ Thus, newer approaches exemplify how protein engineering may broaden the therapeutic toolkit, particularly in difficult-to-penetrate tissues.

### IL-23 inhibitors (IL-23i)

There are 3 monoclonal antibodies targeting IL-23 approved for the treatment of PsA. Ustekinumab, the oldest, blocks the shared p40 subunit of IL-12 and IL-23 with moderate efficacy for skin, peripheral arthritis, enthesitis and dactylitis. More recently, guselkumab, binds to the p19 subunit of IL-23 and was approved for PsA treatment following the DISCOVER trials. Guselkumab also demonstrated significant reduction in radiographic progression. The more recent COSMOS trial, which evaluated patients with an inadequate response to TNFi, confirmed that guselkumab provides sustained improvements in both skin and joint outcomes. These benefits were consistent across all patient subgroups, regardless of baseline characteristics such as sex, obesity, disease duration, and disease severity, which are known to negatively affect outcomes and were observed across various composite measures of disease activity.^45,46^

Risankizumab, the third IL-23i approved for PsA treatment, also specifically inhibits IL-23 by binding to the p19 subunit. Approval followed the KEEPSAKE trials which demonstrated efficacy up to 52 weeks. The trials are ongoing with a recent post hoc analysis looking at the efficacy of risankizumab, in clinically relevant subgroups of patients defined by age, sex, body mass index, race and disease characteristics at weeks 24 and 52. Results showed efficacy in all the risankizumab, treated groups regardless of disease characteristics and prior or concomitant drug use.^47^ Risankizumab, efficacy was also demonstrated across key GRAPPA-recognised domains of PsA over 100 weeks of treatment, with improvements in all the assessed PsA domains. Importantly, the low levels of disease activity achieved across all domains were sustained through 100 weeks of risankizumab, treatment, and new cases of IBD and uveitis were rare.^48^

### Targeted synthetic DMARD (tsDMARD)

#### Janus kinase inhibitors (JAKis)

The advent of JAKis has transformed the management of PsA through their efficacy in all PsA domains, rapid clinical effects and oral administration, adding further to individualised treatment strategies. JAKis modulate intracellular signalling pathways critical to multiple pro-inflammatory cytokines allowing them to influence diverse clinical manifestations, including peripheral arthritis, skin disease, enthesitis, dactylitis and axial involvement.^49^ The convenience of oral dosing, coupled with relatively short half-lives, enables flexible titration and rapid intervention in the event of adverse events, distinguishing JAKis from injectable biologics.

Pivotal evidence for the efficacy of JAKis in PsA came initially from the OPAL Broaden and SELECT-PsA 1 and 2 studies. Tofacitinib, the first approved JAKi for PsA through the OPAL trials, specifically inhibits JAK1 and JAK3. In the OPAL Broaden trial, TNFi–naïve patients with active PsA and an inadequate response to csDMARDs that received tofacitinib demonstrated significantly greater ACR20 response rates and improvements in physical function than placebo.^50^ The OPAL Balance long-term extension study from the OPAL program, provided up to 36 months of efficacy and 48 months of safety data. Sustained improvements were reported across all PsA domains, with a safety profile characterised by low rates of serious infections, malignancies and major adverse cardiovascular events supporting the use of tofacitinib as a first-line option in patients with an inadequate response to csDMARDs and highlighting its benefit for early intervention in disease management.^51^ Upadacitinib a selective JAK1 inhibitor, through the SELECT-PsA 1 and 2 trials demonstrated efficacy in PsA patients with insufficient response to csDMARDs or bDMARDs respectively.^52^ Both trials exhibited significant response rates for Upadacitinib, maintaining efficacy through two years. In SELECT-PsA 1, Upadacitinib outcomes were comparable or superior to adalimumab, including sustained inhibition of radiographic progression. A post hoc analysis looking at enthesitis, often associated with poorer long-term outcomes, showed rapid and significant improvements.^53^ Collectively, these findings confirm the ability of JAKis to provide durable and broad-spectrum efficacy across PsA manifestations.

Newer JAKis include Deucravacitinib and Zacositinib both inhibiting TYK2. Deucravacitinib, an oral selective TYK2 inhibitor, has demonstrated efficacy in patients with moderate-to-severe plaque psoriasis. In the Phase 3 POETYK PSO-1 and PSO-2 trials, deucravacitinib was superior to both placebo and apremilastat week 16, achieving higher rates of PASI 75 and sPGA 0/1 responses.^54,55^ The long-term extension of this study is currently evaluating durability of response and long-term safety. Although performed in psoriasis, these results are highly relevant for PsA, as apremilast remains a widely used oral option.

Zacositinib, a highly selective and potent allosteric TYK2 inhibitor, has also emerged as a promising oral therapy. In phase 2 studies, zacositinib showed rapid and robust improvements in joint and skin endpoints, with a favourable safety profile. Its oral route and targeted cytokine modulation may benefit patients who are refractory to conventional biologics or have contraindications to injectable therapies.^56^ A Phase 3 study named NCT06671483 aims to assess the efficacy and safety of zasocitinib over 60 weeks. Participants with inadequate responses to csDMARDs or NSAIDs, will provide further insight into the therapeutic potential of oral TYK2 inhibition in bDMARD-naive PsA patients.^57^

Additional insights from network meta-analyses comparing various JAKi have enhanced our understanding of differences in efficacy within the class. Across joint, skin, enthesitis and dactylitis measures, most JAKis significantly outperformed placebo. Upadacitinib at 30 mg, consistently demonstrated the strongest efficacy, including superiority over tofacitinib 5 and 10 mg and upadacitinib 15 mg for ACR70 responses and superior resolution of dactylitis compared with deucravacitinib 6 mg.^58^ PASI75 responses and functional improvements (HAQ-DI) were also highest with upadacitinib 30 mg. However, only Upadacitinib 30mg was associated with significantly more serious adverse events versus placebo in the analysis, emphasising the need to balance potency with safety.^58^ Further meta-analyses have offered insights into the comparative efficacy of JAKis. A 2024 systematic review evaluated biologic-naïve PsA patients across multiple targeted therapies, including TNFi, IL-17i, IL-23i, phosphodiesterase 4 inhibitors (PDE4i), and JAKis. The analysis focused on ACR20/50/70 responses, enthesitis and dactylitis resolution, functional improvement and composite joint-skin endpoints (ACR50 plus PASI100). JAKis consistently ranked highest for articular outcomes, outperforming TNFi and IL-17i in joint improvements but their efficacy in extra-articular domains was less pronounced. IL-17i showed superior outcomes in enthesitis and dactylitis resolution, achieved greater physical function improvements and generated higher composite joint–skin responses. Safety profiles varied across therapies: PDE4 inhibitors were associated with the fewest adverse events, whereas JAKis carried a higher overall burden for class-specific risks such as herpes zoster, cytopenias, and thromboembolic or cardiovascular complications. These data reinforce the potency of JAKis as first-line agents in articular disease in biologic-naïve patients, while highlighting limitations in extra-articular domains and the need for careful monitoring.^59^ Nonetheless, the absence of head-to-head comparisons among JAKis limits definitive conclusions about efficacy within the class. While efficacy is well established in bDMARD-experienced populations, their role in biologic-naïve patients is debated. Current EULAR recommendations place JAKis in patients who have failed csDMARDs or biologics, citing long-term safety considerations.^60^ In contrast, GRAPPA guidelines offer greater flexibility, considering JAKis alongside biologics even in DMARD-naïve patients with peripheral arthritis and as valid options for other domains.^61^

JAKis are highly effective, rapidly acting oral therapies providing durable control of articular disease in PsA. While their efficacy is more apparent in joint outcomes, variable intra-class potency and safety considerations necessitate careful patient selection, close monitoring and further studies to optimise their placement within evolving treatment algorithms.

#### Apremilast

Apremilast, an oral small molecule that inhibits PDE4i has shown efficacy in PsA through the PALACE trials.^62–65^ EULAR places it for patients with mild disease and an inadequate response to csDMARDs or when a bDMARDs or a JAKi is appropriate. This is due to a relatively low efficacy and lack of data on inhibition of damage progression, as well as little evidence on its efficacy on enthesitis resolution.^60^ To prevent structural damage, early suppression of inflammation is important, as shown previously and further confirmed by the recent TICOPA post hoc analysis of radiographic progression.^66^ This data confirmed the benefit of achieving low disease activity on subsequent radiographic outcomes in patients with PsA. Magnetic resonance imaging (MRI) is a highly sensitive tool for detecting peripheral inflammation and bone erosion. Methods have been developed to evaluate its use in PsA. The Psoriatic Arthritis Magnetic Resonance Imaging Scoring System (PsAMRIS) of the hand and the MRI Whole-Body Scoring System for Inflammation in Peripheral Joints and Entheses in Inflammatory Arthritis (MRI-WIPE), enable assessment of inflammation in joints and entheses of the entire body at even earlier stages than plain radiography. The recently published MOSAIC study evaluated the impact of apremilast treatment on inflammation, with outcomes measured by PsAMRIS and MRI-WIPE. Despite limitations, results showed that apremilast improved inflammation in both joints and entheses when using MRI. The MOSAIC study also provided important information into the value of PsAMRIS and MRI-WIPE as objective measures in PsA, supporting their potential use as sensitive tools to measure treatment response in PsA patients.^67^ Beyond joint and skin involvement, PsA is also associated with metabolic and cardiovascular comorbidities. For example, a recent cross-sectional study demonstrated that patients with PsA exhibited significantly higher rates of hypertension, hyperlipidaemia, and diabetes compared with patients with psoriasis alone.^68^ Interestingly, apremilast is known to cause weight loss. The Immune Metabolic Associations in Psoriatic Arthritis study showed that apremilast treatment was associated with significant reductions in body weight and abdominal subcutaneous fat, along with improvements in psoriatic disease activity that were independent of weight change. However, no significant changes were observed in glucose parameters, lipid profiles, or vascular function markers such as endothelial function, suggesting that the benefits of apremilast are likely driven by direct immunological mechanisms rather than broader cardiometabolic effects.^69^ A more recent prospective cohort study examining apremilast’s effects on body weight, body composition and cardiovascular risk factors in PsA confirmed the significant reduction in total and android fat mass and improvements in disease activity, while showing no significant changes in blood pressure, lipid or glucose levels. The beneficial cardiovascular and metabolic effects along with the reduced android fat mass correlated with improved disease activity, suggesting that apremilast may have beneficial cardiovascular and metabolic effects by reducing fat mass and potentially lowering the risk of cardiovascular events.^70^

#### Combination treatments in PsA

The use of combination regimens with methotrexate (MTX) plus bDMARD have improved bDMARD survival in rheumatoid arthritis, but their use in PsA remains controversial. A current PsA review, where MTX was added to a bDMARD, showed similar outcomes between combination and monotherapy.^71^ The National Ambulatory Medical Care Survey data on PsA treatment patterns also showed the use of traditional combination therapy declining after 2013, reflecting the improved efficacy of newer biologics.^72^ Yet, a current meta-analysis illustrated significantly better bDMARD drug survival with combination therapy versus bDMARD monotherapy especially for TNFis.^73^

Combination treatment regimens with two bDMARDs may be particularly relevant in patients with difficult to treat (D2T) PsA or with overlapping disease manifestations, as in PsA with coexisting uveitis or IBD, where different pathogenic pathways may drive different domains of the disease. Nevertheless, the use of two bDMARDs raises concerns about safety, particularly infection risk, highlighting the need for further data to clarify risk-benefit. A multi-centre audit in the UK revealed limited use of combination bDMARD regimens for PsA. These were used predominantly for patients who had failed previous biologics. Initial response rates were good, and treatment persistence was high, with no increased reports of serious adverse events or infections.^74^ A recent systematic literature review on the use of two different b- or ts-DMARDs in immune-mediated inflammatory diseases showed the most frequent combinations were TNFi/IL-23i, JAKi/bDMARD, and vedolizumab/TNFi combinations with 50% of patients showing benefits without significant safety issues.^75^

#### Difficult to treat PsA

Despite the progress in PsA management, a significant number of patients don’t achieve MDA or remission. The management of D2T PsA remains a significant challenge in modern rheumatology. While GRAPPA has recently established consensus definitions, their clinical application is still evolving. The persistence of symptoms despite failing three or more treatments with different mechanisms of action, including two b or tsDMARDs with objective evidence of ongoing inflammation is considered D2T. However not all patients labelled D2T have refractory inflammation. The recently proposed complex-to-manage PsA profile highlights that many of these patients present a complex combination of comorbidities, psychosocial factors and pain mechanisms that complicate treatment response.^76.^ The challenge is distinguishing these patients from those with inflammatory pain, as misclassification may lead to unnecessary treatment escalation.^77^ For patients with confirmed active inflammation, shifting to a drug with a different mechanism of action is essential. The CorEvitas PsA/SpA Registry results showed continued cycling within the same class to be ineffective.^78^ Better phenotyping with the help of imaging, particularly musculoskeletal ultrasound, can reveal subclinical inflammation and potentially aid early stratification.^79^ This approach though, requires prospective studies for validation. Furthermore, combining bDMARDs as previously mentioned or dose intensification are off-label promising strategies also requiring further validation in this patient group.^80^ Simultaneously the management of non-inflammatory factors (such as weight reduction, physical therapy or exercise and management of chronic pain) may also improve outcomes.^81^ There are no randomised trials yet specifically for D2T PsA. Most guidance extrapolates from broader PsA populations. To move forward a consensus on case definition and the development of trials tailored to D2T patients is needed. Only then can true evidence-based treatment strategies and outcomes be tested in this complex and heterogeneous population.

## CONCLUSION

Over the past year, advances in PsA research have deepened our understanding of disease mechanisms, improved early recognition of the PsO–PsA transition and expanded therapeutic options across all clinical domains. Novel therapies and evolving treatment algorithms allow for more individualised approaches, while newer insights into pathogenesis are driving progress toward precision medicine. At the same time, challenges such as gender-specific responses, comorbidity burden and the management of D2T PsA underscore the complexity of this disease. Looking ahead, integrating biomarkers, imaging and personalised treatment strategies into routine practice will be key to achieving sustained remission, preventing progression and improving quality of life for patients living with PsA.
